# Supplementary material for: Spontaneous mutations in hlyD and tuf genes result in resistance of Dickeya solani IPO 2222 to phage ϕD5 but cause decreased bacterial fitness and virulence in planta
Source: Sci Rep. 2023 May 9;13:7534. doi: 10.1038/s41598-023-34803-7 (PMC10169776; doi:10.1038/s41598-023-34803-7)

*IFB running title:* Spontaneous ϕD5-resistant *Dickeya solani* mutants

**Supplementary Materials for:**

**Spontaneous mutations in *hlyD* and *tuf* genes result in resistance of *Dickeya solani* IPO 2222 to phage ϕD5 but cause decreased bacterial fitness and virulence *in planta***

Daryna Sokolova ^1, 2^, Anna Smolarska ^3^, Przemysław Bartnik ^1^, Lukasz Rabalski ^4^, Maciej Kosinski ^4^, Magdalena Narajczyk ^5^, Dorota M. Krzyzanowska ^1^, Magdalena Rajewska ^6^, Inez Mruk ^7^, Paulina Czaplewska ^7^, Sylwia Jafra ^6^ and Robert Czajkowski ^1^*

^1^ Laboratory of Biologically Active Compounds, Intercollegiate Faculty of Biotechnology UG and MUG, University of Gdansk, A. Abrahama 58, 80-307 Gdansk, Poland

^2^ Department of Biophysics and Radiobiology, Institute of Cell Biology and Genetic Engineering, National Academy of Sciences of Ukraine, 148 Academika Zabolotnoho St., 03143, Kyiv, Ukraine

^3^ Department of Cancer Biology, Institute of Biology, Warsaw University of Life Sciences (SGGW), J. Ciszewskiego 8, 02-786 Warsaw, Poland

^4^ Laboratory of Recombinant Vaccines, Intercollegiate Faculty of Biotechnology UG and MUG, University of Gdansk, A. Abrahama 58, 80-307 Gdansk, Poland

^5^ Laboratory of Electron Microscopy, Faculty of Biology, University of Gdansk, Wita Stwosza 59, 80‐308 Gdansk, Poland

^6^ Laboratory of Plant Microbiology, Intercollegiate Faculty of Biotechnology UG and MUG, University of Gdansk, A. Abrahama, 58, 80-307 Gdansk, Poland,

^7^ Laboratory of Mass Spectrometry‐Core Facility Laboratories, Intercollegiate Faculty of Biotechnology UG and MUG, University of Gdansk, Antoniego Abrahama 58, 80‐307 Gdansk, Poland

* To whom correspondence should be addressed: Robert Czajkowski, Laboratory of Biologically Active Compounds, Intercollegiate Faculty of Biotechnology UG and MUG, University of Gdansk, Antoniego Abrahama 58, 80-307 Gdansk, Poland, phone: 0048 58 5236333, e-mail: robert.czajkowski@ug.edu.pl

**Supplementary Table 1.** Differencing phenotypes of the phage-resistant mutants DsR34 and DsR207 compared to the phenotypes of *D. solani* IPO 2222 WT screened with BIOLOG phenotypic microarray GENIII, EcoPlate, PM1, and PM2a plates.

| **compound** | **IPO 2222 WT** | **Mutant DsR34** | **Mutant DsR207** |
| --- | --- | --- | --- |
| D-cellobiose | + | - | - |
| D-turanose | + | - | - |
| 4% NaCl | + | - | - |
| gentiobiose | + | - | - |
| N-acetyl-*β*-D-mannosamine | - | + | + |
| L-glutamic acid | - | + | + |
| inulin | - | + | + |
| D-tagatose | - | + | + |
| malonic acid | - | + | + |

**+** - the ability to utilize the mentioned compound for growth

**-** - the inability to utilize the mentioned compound for growth

**Supplementary Figure 1.** Ability of ΦD5-resistant *D. solani* mutants DsR34 and DsR207 to cause maceration (rotting) of potato tuber tissue. Quantitative determination of the average weight of the rotting tuber tissue (in grams) collected after 72 h incubation at 28 ºC under humid conditions. Per mutant, five individual potato tubers were inoculated in two independent experiments (n=10). IPO 2222 wild-type strain was used as a positive control. Tubers inoculated with sterile demineralized water served as the negative control. Results were considered significant at p=0.05, and pairwise differences were acquired with the use of the t-test. The means not sharing the same letters above each bar vary. Error bars represent standard deviation (SD).

**
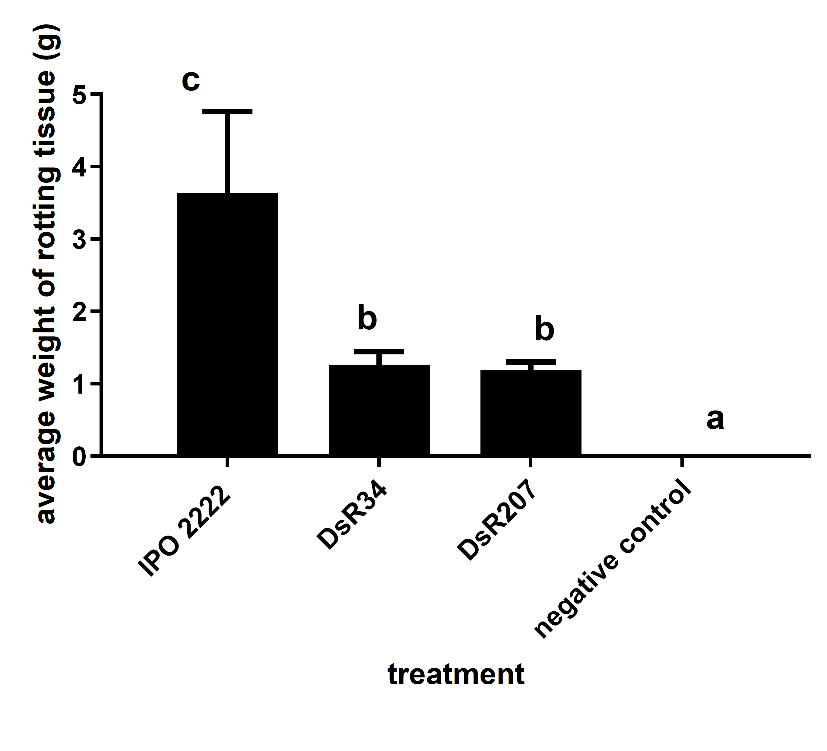
**

**Supplementary Figure 2.** Ability of ΦD5-resistant *D. solani* mutants DsR34 and DsR207 to cause maceration (rotting) of chicory leaves. Quantitative determination of the average rotting area (in mm2) after incubation at 28 ºC in a humid box. Per mutant, five individual chicory leaves were inoculated in two independent experiments (n=10). Results were considered significant at p=0.05, and pairwise differences were obtained using the t-test. The means not sharing the same letters above each bar vary. Error bars represent standard deviation (SD).

**
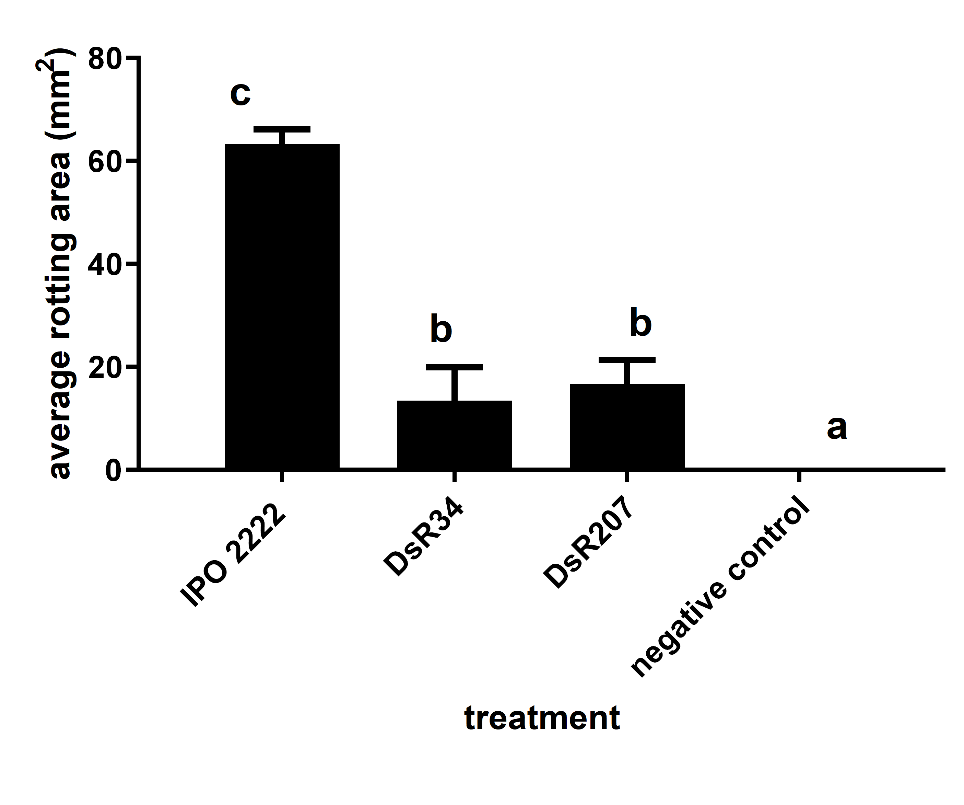
**

**Supplementary Figure 3.** Ability of ΦD5-resistant *D. solani* mutants DsR34 and DsR207 to colonize and cause symptoms in potato plants cv. Kondor grown in potting compost under phytochamber conditions. Plants were grown for 14 days for rooting and shoot developments. Rooted and developed plants were inoculated with D. solani IPO 2222 wild-type strain or ΦD5-resistant DsR34 and DsR207 mutants (n=10 per treatment) by application of 50 ml of bacterial suspension (ca. 108 CFU ml-1) in sterile Ringer's buffer directly to the soil surrounding stem bases of each plant. As a negative control, the soil was treated with sterile Ringer's buffer instead of the bacterial suspension. After two weeks, the samples were inspected for symptoms and bacterial populations inside stems. **A** – Percentage of symptomatic plants 14 days post inoculation (DPI) in both experiments. **B** – Population size of bacterial strains within stems of potato plants after introducing the pathogen into the soil in both experiments. Results were considered significant at p = 0.05, and the pairwise differences were obtained using the t-test. The means not sharing the same letters above each bar vary. Error bars represent standard deviation (SD).

**
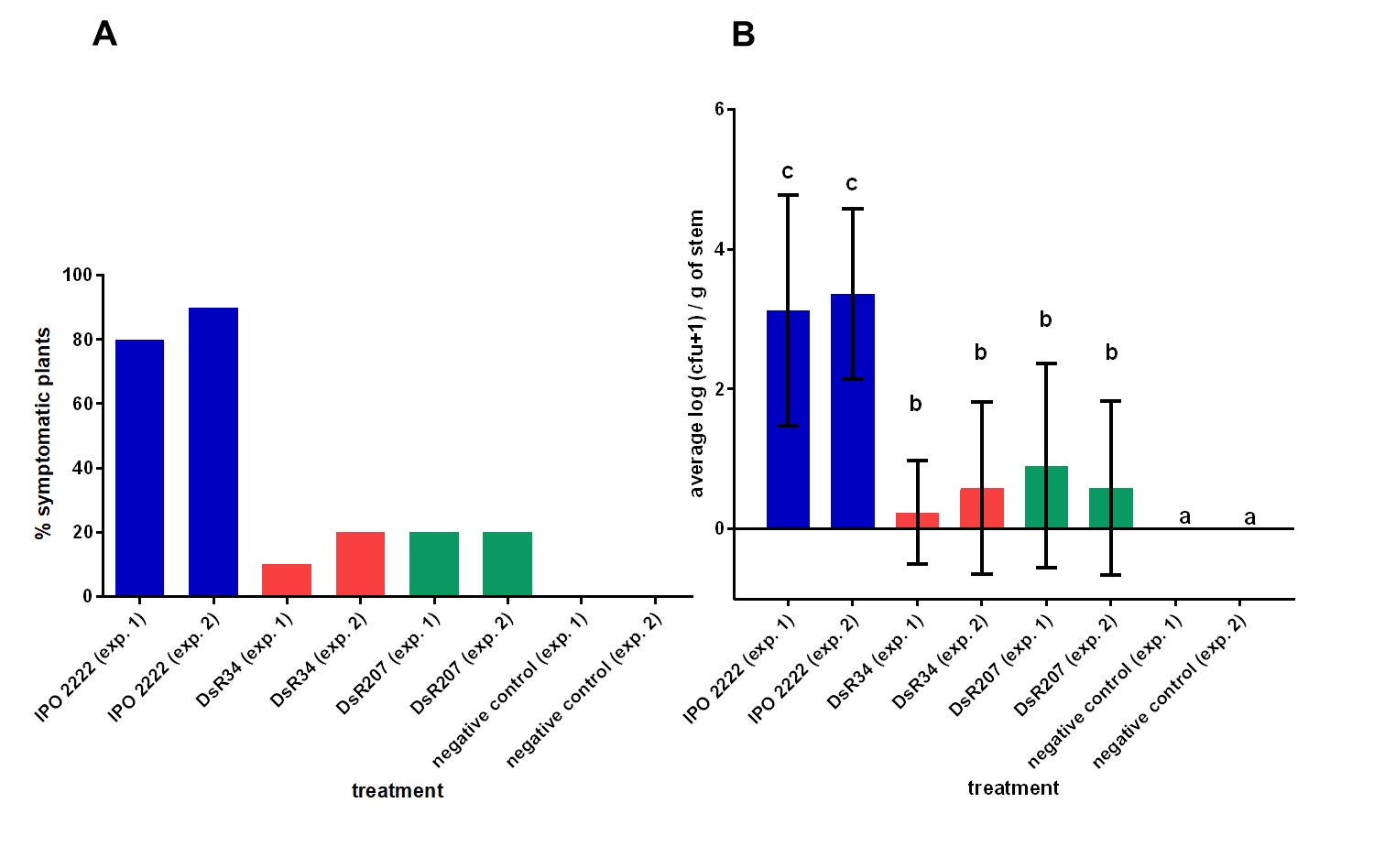
**

**Supplementary Figure 4.** Self-aggregation of cells of *D. solani* IPO 2222 wild-type strain and phage-resistant DsR34 and DsR207 mutants as measured by turbidity of the bacterial suspension. The percentage of aggregation was quantified from the change in optical density (OD600) over 24 h. Percentage aggregation (sedimentation) was measured as follows: %A = 1-( OD600 24h/OD600 0h), where: %A—the percentage of aggregation (sedimentation), OD600 0h—OD of bacterial culture at time 0 h, OD600 24h— OD of bacterial culture at time 24 h. Results were considered significant at p = 0.05, and pairwise differences were obtained using the t-test. The means not sharing the same letters above each bar vary. Error bars represent standard deviation (SD).

**
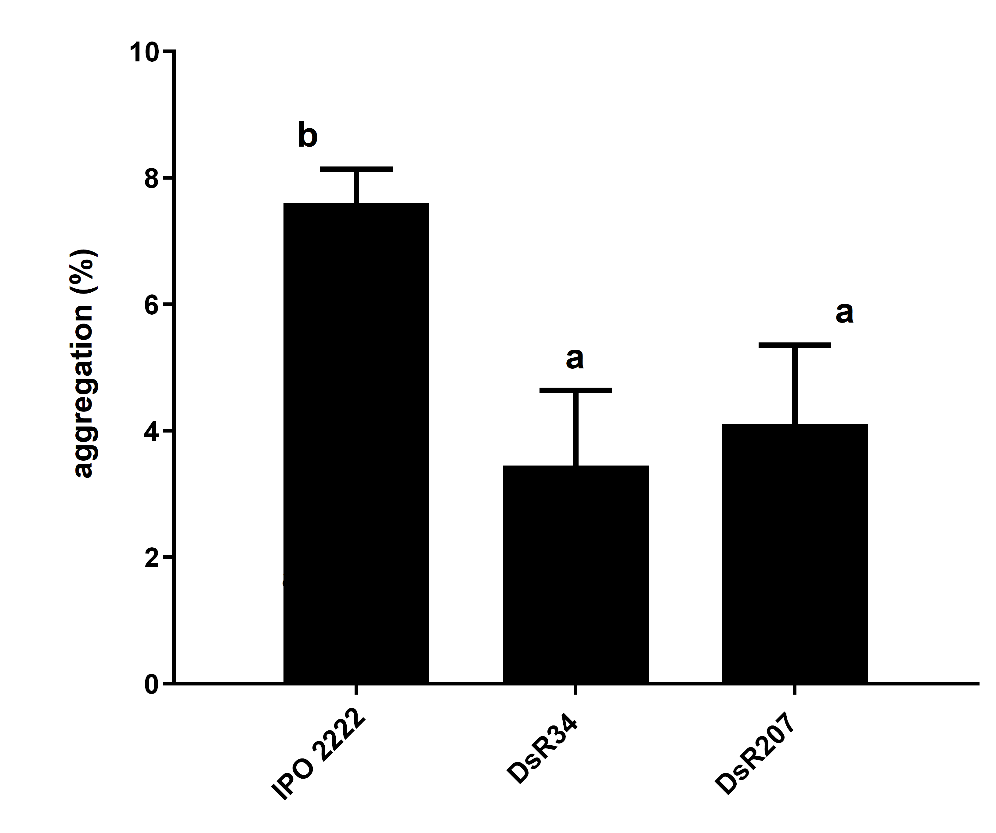
**

**Supplementary Figure 5.** Characterization of lipopolysaccharide (LPS) from *D. solani* IPO 2222 wild-type strain and phage-resistant DsR34 and DsR207 mutants. SDS‐PAGE was done using a gradient (4–20%) polyacrylamide gel, and the LPS components were visualized by silver staining 83. The kDa marker (11–245 kDa, Perfect Tricolor Protein Ladder, EURx, Poland) is shown in the first lane. * - marks band present in the wild-type strain but absent in phage-resistant DsR34 and DsR207 mutants.

**
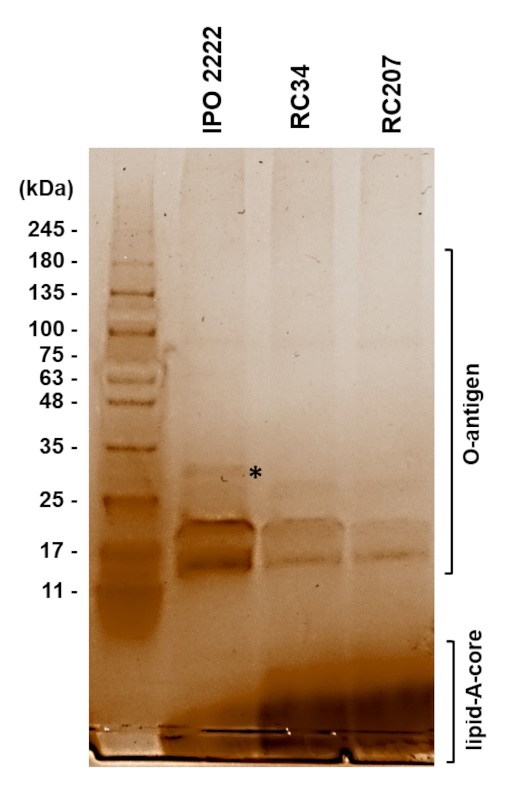
**

**Supplementary Figure 6.** Proteomes of phage-resistant *D. solani* mutants DsR34 and Dsr207. A, C – Venn plot illustrating protein identification overlaps between the wild-type IPO 2222 strain and ΦD5-resistant mutants DsR34 and DsR207, B, D - Significantly differentially abundant proteins related to the envelope biogenesis, metabolism, and regulation in phage-resistant mutants DsR34 and DsR207 and the wild-type IPO 2222.


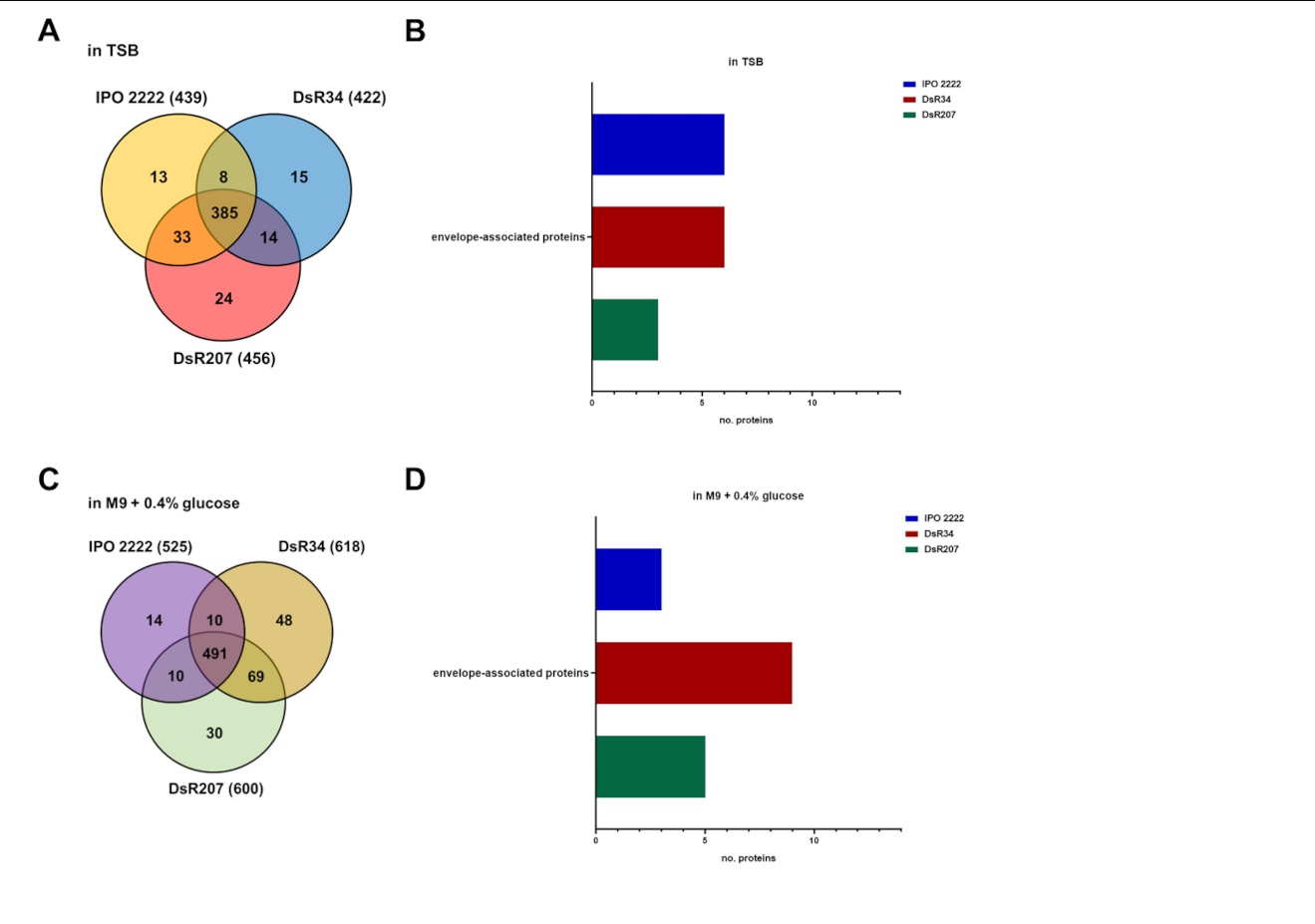


**Supplementary Figure 7.** Growth of *D. solani* IPO 2222 wild-type strain and phage-resistant DsR34 and DsR207 mutants in Tryptone Soya Broth (TSB) supplemented with 0.1% EDTA. The experiment was done using two biological replicates containing two technical replicates each (n=4). The results were averaged for presentation. The bars show the standard deviation (SD).

**
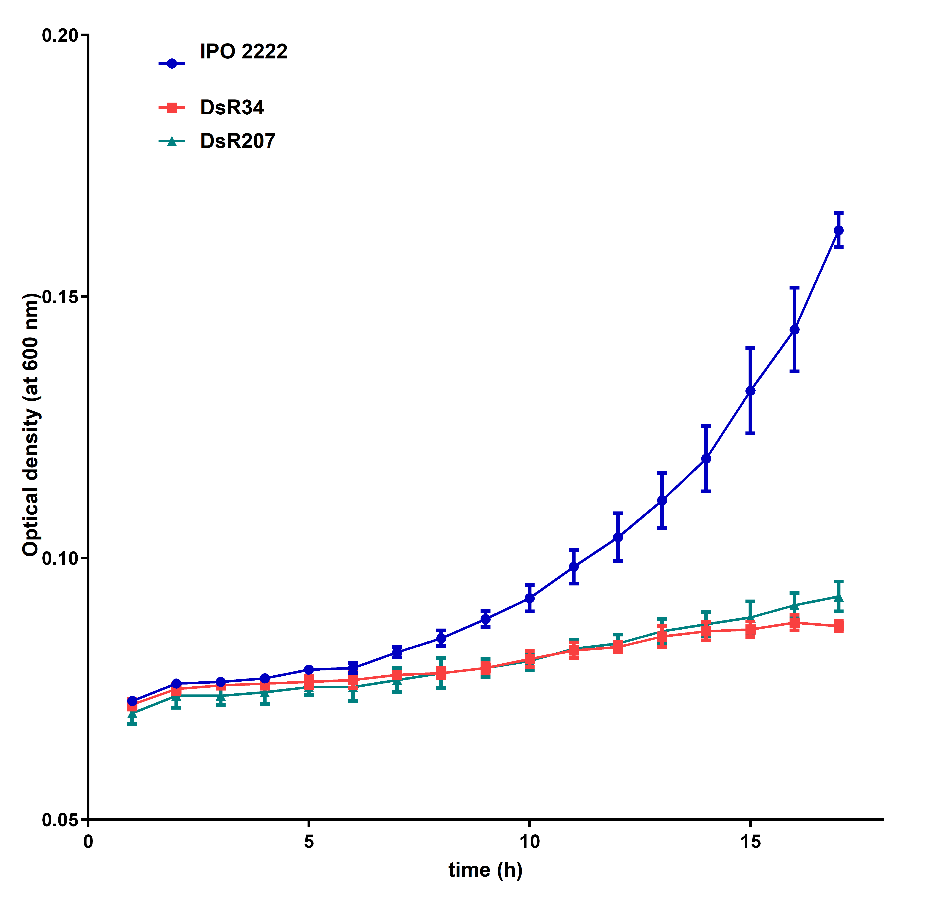
**

**Supplementary Figure 8.** Function category of significantly differentially abundant proteins in phage-resistant mutants DsR34 and DsR207 and the wild-type IPO 2222 in TSB (rich medium) (**A**) and M9+glucose (minimal medium) (**B**)


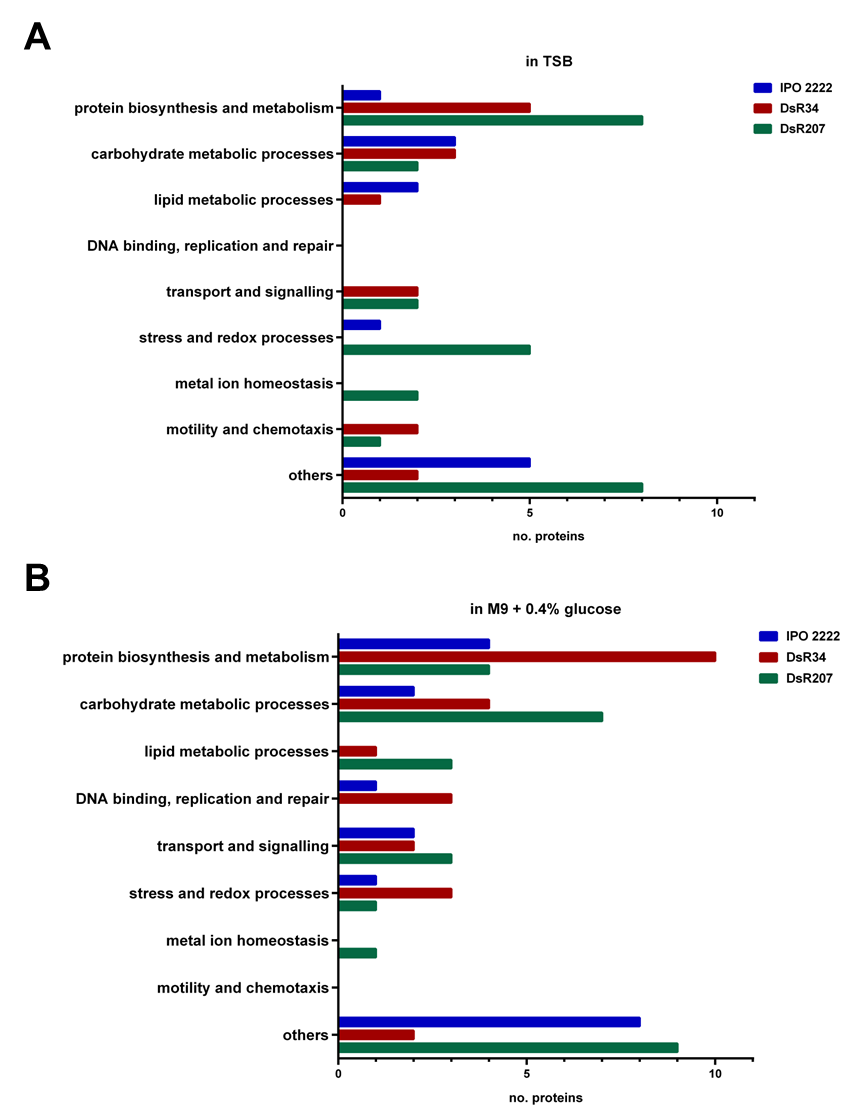

Supplement: Supplementary file 1 — Supplementary Information. [file 41598_2023_34803_MOESM1_ESM.docx]
